# Chagas Disease: From Discovery to a Worldwide Health Problem

**DOI:** 10.3389/fpubh.2019.00166

**Published:** 2019-07-02

**Authors:** Kárita Cláudia Freitas Lidani, Fabiana Antunes Andrade, Lorena Bavia, Flávia Silva Damasceno, Marcia Holsbach Beltrame, Iara J. Messias-Reason, Thaisa Lucas Sandri

**Affiliations:** ^1^Laboratory of Molecular Immunopathology, Clinical Hospital, Federal University of Paraná, Curitiba, Brazil; ^2^Laboratory of Biochemistry of Tryps-LaBTryps, Department of Parasitology, Institute for Biomedical Sciences, University of São Paulo, São Paulo, Brazil; ^3^Laboratory of Human Molecular Genetics, Department of Genetics, Federal University of Paraná, Curitiba, Brazil; ^4^Institute of Tropical Medicine, University of Tübingen, Tübingen, Germany

**Keywords:** Chagas disease, epidemiology, *Trypanosoma cruzi*, Chagas cardiomyopathy, neglected disease

## Abstract

Carlos Chagas discovered American trypanosomiasis, also named Chagas disease (CD) in his honor, just over a century ago. He described the clinical aspects of the disease, characterized by its etiological agent (*Trypanosoma cruzi*) and identified its insect vector. Initially, CD occurred only in Latin America and was considered a silent and poorly visible disease. More recently, CD became a neglected worldwide disease with a high morbimortality rate and substantial social impact, emerging as a significant public health threat. In this context, it is crucial to better understand better the epidemiological scenarios of CD and its transmission dynamics, involving people infected and at risk of infection, diversity of the parasite, vector species, and *T. cruzi* reservoirs. Although efforts have been made by endemic and non-endemic countries to control, treat, and interrupt disease transmission, the cure or complete eradication of CD are still topics of great concern and require global attention. Considering the current scenario of CD, also affecting non-endemic places such as Canada, USA, Europe, Australia, and Japan, in this review we aim to describe the spread of CD cases worldwide since its discovery until it has become a global public health concern.

## Introduction

Chagas disease (CD) is an anthropozoonosis caused by the protozoan parasite *Trypanosoma cruzi*, that affects about 6–8 million people worldwide (1) and causes approximately 50,000 deaths per year. Another 65–100 million people are living in areas at risk for infection worldwide (2–4). Even though over a century has passed since its discovery, CD remains one of the leading public health problems for most Latin American countries. In recent decades, CD has also been a concern for non-endemic places such as Canada, USA, Europe, Australia and Japan due to the constant migration of individuals from endemic areas (5). In this situation, the transmission occurs mainly through blood transfusion, organ transplantation or vertical transmission from mother to child.

The infection has two successive phases. The acute phase is characterized by a high parasitemia, usually asymptomatic or oligosymptomatic with fever, anorexia, and tachycardia (6). These manifestations disappear spontaneously in 90% of the cases, and possibly 60–70% of infected individuals will never develop signs or symptoms related to CD, characterizing the indeterminate form. The remaining patients (30–40%) may progress to the chronic phase with neurological, cardiac, digestive (megacolon or megaesophagus), or cardiodigestive clinical complaints (7). Chronic chagasic cardiomyopathy (CCC) is the most serious manifestation of the disease, affecting one-third of individuals with positive serology (8), and in severe cases, the only treatment option is heart transplantation. Despite efforts to understand the parasitic tropism for certain tissues, such as the heart, the factors involved in the clinical progression from indeterminate to symptomatic forms are still unknown.

Chronic CD is considered a disabling disease responsible for the most significant morbidity and mortality among parasitic diseases (9), leading to a global expenditure of USD$627.5 million per year in health care costs (10). The estimated cost per patient at the early stages of the disease is $200, but in the chronic symptomatic form, this value can reach 4,000 to 6,000 dollars (11). Considering that the current scenario of CD is changing to also affecting non-endemic countries, in this review we aimed to describe the spread of CD cases worldwide from its discovery to its current status as a global public health concern.

## Discovery of Chagas Disease

CD was named in honor of its discoverer, Carlos Ribeiro Justiniano Chagas (12), who was born on a coffee farm at Oliveira, Minas Gerais state, in Brazil, on July 9th, 1878 (13). Chagas graduated in Medicine in 1903 and was invited by Oswaldo Cruz to work as a physician at the Ministry of Public Health and Hygiene in Brazil, where he first applied the intra-household vector control against malaria. Due to his success in his work, Chagas became a member of the National Academy of Medicine of Brazil and received several awards and titles from institutions in Paris, Belgium, Lima, and the US, including Doctor Honoris Causa from Harvard University. Besides Chagas was nominated twice for the Nobel Prize in Medicine and Physiology (1913 and 1921), but for unclear reasons, he was never awarded (13). Some evidence points toward political opposition to Chagas in Brazil, due to the socio-economic feature of the disease (14, 15). Furthermore, researchers from Europe did not accept this unusual discovery (15, 16), and Chagas disease was still not been completely understood by 1912. In 1921, although Chagas had established the principal characteristics of the new disease and published it in a relevant journal of the time, surprisingly there was no written report about the Chagas evaluation in the Nobel Committee of Karolinska Institute, and no scientist received the prize that year (16, 17). He headed the Oswaldo Cruz Institute for 17 years (from 1917 until his death in 1934) and coordinated a campaign against the Spanish flu epidemic in Brazil (1918).

**Figure 1:**
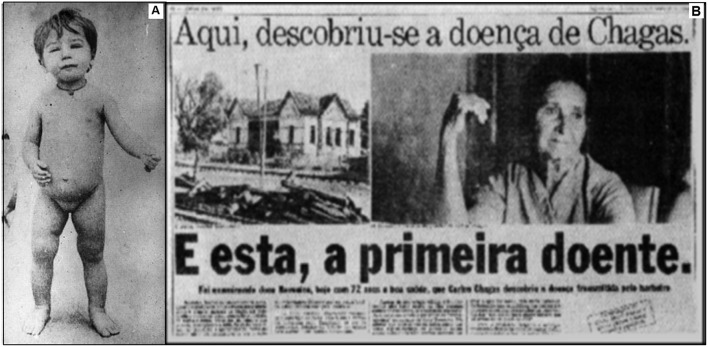
**(A)** Berenice: the first patient diagnosed with Chagas disease. **(B)** Brazilian newspaper reporting the history of Chagas disease and Berenice, saying “Chagas disease was discovered here” (up), and: “And this is the first case” (down). Source: Archives of the Instituto Oswaldo Cruz.

On February 14th, 1909, Chagas consulted a patient that would be the first CD case described in the literature: a 2-year-old child, Berenice (Figure 1), who had a high fever, hepatosplenomegaly, face edema and presence of the parasite in the blood (12). Berenice remained asymptomatic throughout her life and died at 73 years from other causes. She was included in several clinical studies of CD from the age of 55 to 71 years old.

**Figure 2:**
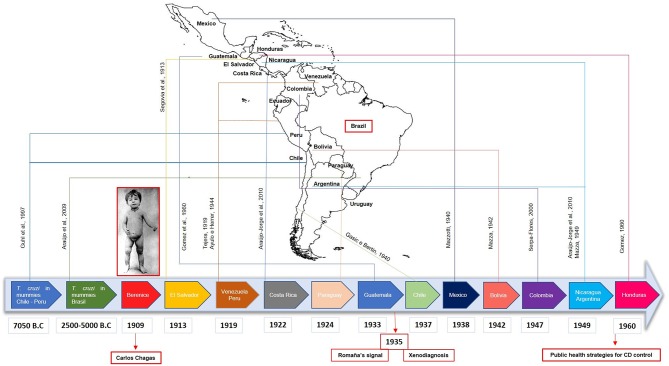
Timeline with the first human cases of Chagas Disease (CD) reported in Latin America. Following the report of the first patient confirmed with CD (Berenice) in Brazil by Carlos Chagas in 1909, cases were reported in several countries such as El Salvador, Venezuela, Peru, Costa Rica, Paraguay, Guatemala, Chile, Mexico, Bolivia, Colombia, Nicaragua, Argentina, and Honduras, with essential findings in paleoparasitology field. In 1935, the Romaña signal was associated with the acute form of CD and the xenodiagnosis was proposed. Only in 1960, government programs were implemented to control CD in Latin America.

Following this, the investigation on CD in Latin America was intensified, with the first reports of the disease in 1913 in El Salvador (18); in 1919 in Peru (19) and in Venezuela (20); in 1922 in Costa Rica (21); in 1924 in Paraguay (21); in 1933 in Guatemala (22); in 1937 in Chile (23); in 1938 in Mexico (24); in 1942 in Bolivia (25); in 1947 in Colombia (26); in 1949 in Nicaragua (21) and in Argentina (27); and 1960 in Honduras (22). More recently, *T. cruzi* DNA has been found in mummies from Chile/Peru (28) and Brazil, dating from 7,050 years B.C. and 2,500–5,000 years B.C., respectively, demonstrating that the disease has existed in Latin America for more than 9,000 years (29, 30) (Figure 2). Despite dating to the pre-Columbian period, CD has not been mentioned before 1909, which makes the findings of Carlos Chagas a unique achievement in the history of parasitology and medicine. He alone described the most important features of a new tropical disease: the vector, the pathogen and its different stages of development, the hosts, as well as its clinical manifestations, epidemiology and even the prophylaxis of the disease.

According to Lannes-Vieira et al. (31), the history of CD can be divided into three important phases. The first from 1909 to 1934, is characterized by the brilliant work of Chagas and the controversy regarding the definition and legitimation of the disease as a scientific fact and social problem. The second phase, from 1935 to 1960, falls after the death of Chagas when Mazza and Romaña confirmed the acute form of the disease in Argentina and when Evandro Chagas (Chagas' son) and Emmanuel Dias identified the endemic and chronic character of the disease. Finally, the third phase, from 1961 to present day represents a challenge for both the control and the understanding of CD in diverse aspects, in which the implementation of national and international health policies became a constant need.

## Chagas Disease

Also known as American trypanosomiasis, CD is caused by the protozoan parasite *Trypanosoma cruzi*. With complex pathophysiology and a dynamic epidemiological profile, CD remains an important public health concern and is an emerging disease in non-endemic countries.

### *Trypanosoma cruzi* and Vector

*T. cruzi* is a hemoflagellate intracellular parasite that belongs to the order Kinetoplastida, family Trypanosomatidae (32). It is the causative agent of CD, possessing the capacity to infect any cell, mainly macrophages, fibroblasts and epithelial cells (33). During its life cycle, the parasite evolves through three main forms: amastigotes (proliferative form found inside vertebrate host cells), epimastigotes (proliferative form found in intestine of invertebrate hosts), and trypomastigotes (the infective form originated from amastigotes in vertebrate hosts; and from epimastigotes in the digestive tract of invertebrate hosts) (34).

CD is a vector-borne disease, being the parasite transmitted by blood-sucking bugs (also known as “kissing-bug”) from subfamily Triatominiae (35, 36). It is known that 140 species of Triatominae are capable of transmitting *T. cruzi* and are widely distributed in the Americas (37). The most important vectors species are: *Triatoma infestans* in Argentina, Bolivia, Brazil, Chile, Paraguay, Uruguay and Peru; *Rodhinus prolixus* in Colombia, Venezuela and Central America; *T. dimidiata* in Ecuador and Central America; and *Rhodnius pallescens* in Panama (PAHO). In the southern part of USA, the common insect vector is *Triatoma gerstaeckeri*, followed by *T. lecticularia* and *T. sanguisuga* (38).

### Transmission Routes

The vectorial route is considered the classic mode of *T. cruzi* transmission and the most interesting from an epidemiological point of view, due to its direct connection to social, cultural and economic aspects of a population (39). Interestingly, the endemic area for CD highly overlaps with the distribution of most bugs from the Triatominae family (40). With this, systematic insect control drastically reduces or even eliminates the disease expansion (41, 42). In this sense, several international efforts were done in the last decades and resulted in vectorial control in Central America, Brazil, Uruguay, Chile, Argentina, and Paraguay (42). In the sylvatic cycle, the mammalian reservoirs are mostly primates, rodents, and marsupials. In the domestic cycle, the mammalian reservoirs are humans, dogs, and cats (34).

As there is a considerable flow of migrants from endemic to non-endemic countries, *T. cruzi* transmission by transfusion of contaminated blood poses a barrier to disease control (43). Transfusion transmission depends on technical parameters in the trial for blood donors as systematic screening for anti-*T. cruzi* antibodies, and intrinsic aspects of donor or receptor as parasite load and immune status, respectively (44). Also, the transmission through infected organs needs to be carefully followed up with, since *T. cruzi* infection may become exacerbated due to the immunosuppressive status of the organ recipient (45).

According to the World Health Organization (3), there are 1,124,930 women between 15 and 44 years old infected with *T. cruzi* in Latin America, and the overall risk of congenital *T. cruzi* infection in children born from infected mothers is around 5% (46). The success of maternal-fetal transmission depends mainly on parasite genetic variability and maternal-fetal immune responses (43).

The oral transmission route was observed first in animal models in 1913 (47) when it was proposed that reservoirs could acquire the parasite through feeding of contaminated bugs (48). This was later confirmed by experimental infections in a murine model using contaminated blood with trypomastigotes, urine, and feces from contaminated bugs or cultured *T. cruzi* (49). In humans, oral transmission of *T. cruzi* has been described as point source outbreaks in which groups of people have shared contaminated food or beverages during an event (50). The first cases of CD transmitted by oral route were described in Brazil by Silva et al. (51) in Rio Grande do Sul state, and by Shaw et al. (52) in Pará state, and both occurred due to ingestion of a shared meal (50). After the implementation of eradication measures for the vectors and the establishment of routine tests to detect *T. cruzi* in blood bank trials, the oral route has emerged as an important form of transmission. Additionally, some foodborne cases were reported in regions where triatomine intradomiciliary and peridomestic control has been effective (53).

### Clinical Forms

CD has complex pathophysiology and a variable clinical presentation. After the primary *T. cruzi* infection, the acute phase is characterized by a high-grade parasitemia and is, in most cases, asymptomatic. However, symptomatic manifestations of the acute phase—such as prolonged fever, headache, myalgia, lymphadenitis, hepatomegaly, and splenomegaly—usually cease within 60 days even without the use of etiological drugs (54). In the case of vector transmission, the infected individual can present clinical signs resulting from *T. cruzi* inoculation at the portal of entry: chagoma when the entry occurred through the skin or Romaña's sign when it occurred through the periorbital mucosa (6). After a period of 4 to 8 weeks, the parasitemia decreases and the clinical manifestations spontaneously disappear in 90% of the cases, when the disease enters the chronic phase (55).

During the chronic phase, the infection remains clinically silent for life in 60 to 70% of cases, characterizing the asymptomatic (also known as indeterminate) form of CD (34). Nevertheless, after 10–30 years, 30–40% of the asymptomatic patients will develop clinical manifestations, among them neurological (rare), digestive (megacolon and megaesophagus syndromes), cardiac or cardio digestive (7). Cardiac involvement is the most serious manifestation of CD, affecting 1/3 of infected individuals at some point in their lives (8). Chronic chagasic cardiomyopathy (CCC) is characterized by diffuse myocarditis, with tissue substitution by fibrosis and segmental wall motion abnormality (56), with the dilated cardiomyopathy with heart failure being considered the late stage of clinical progression (55). The digestive form of CD is due to denervation of the enteric nervous system that regulates the motor functions of the digestive tract, which results in dysphagia (57). The hypomotility of the digestive system also leads to a dilatation of the colon with consequent massive constipation (58, 59).

Although in many cases, both megacolon and megaesophagus decrease patient's quality of life, when the concomitant development of CCC occurs, characterizing cardiodigestive form, the prognosis is limited (57).

### Diagnosis and Treatment

The diagnosis of human CD can be performed at any stage of the disease and involves the analysis of clinical, epidemiological, and laboratory data (60). In the acute phase, it is possible to determine the presence of circulating parasites in the peripheral blood by parasitological tests, that can be direct as blood smear or thick blood smear, or by multiplication as hemoculture, xenodiagnoses, and polymerase chain reaction (PCR) (61). In the chronic phase, at least two serological tests based on different principles must be performed to detect anti-*T. cruzi* IgG antibodies, such as indirect immunofluorescence, hemagglutination, and enzyme-linked immunosorbent assay (ELISA). In case of blood trial in blood banks, a single ELISA test is sufficient to decide on blood exclusion (3). In addition to parasitological and serological tests, routine laboratory tests, electrocardiogram (ECG), chest radiography, and hepatogram are requested both in the acute and chronic phases for clinical evaluation (62–64).

CD treatment involves both parasite-specific and symptomatic treatments (65). The drugs currently in use as antiparasitic therapy in CD, benznidazole and nifurtimox, are effective in the acute cases, in congenital cases, and in reactivation due to immunosuppression; however, treatment is often discontinued due to a required prolonged course and various adverse effects (5, 55, 66). Although there is no consensus for the use of the treatment in the chronic phase, studies have shown that antiparasitic treatment was able to prevent the onset or delay the progression of CD in the evaluated cases (62, 67). On the other hand, in a multi-center study, named BENEFIT (Benznidazole Evaluation for Interrupting Trypanosomiasis), patients with CCC were treated with benznidazole and no delay in the clinical progression was observed for the most severe forms of cardiomyopathy (68). However, the treatment reduced the number of associated clinical intercurrences (69). Recently, a clinical trial (BENDITA) demonstrated that changing treatment protocol duration from 8 weeks to 2 weeks with a daily dose of 300mg/day of benznidazole was efficient after completing treatment or at the 12-month follow-up (70).

It is important to emphasize that the cure rate and its confirmation depend on factors such as phase and duration of disease, age of the patient, the tests used for the evaluation of therapeutic efficacy and the time of follow-up after treatment, associated comorbidities, and even the susceptibility of the *T. cruzi* genotype to the anti-parasitic drugs used (71).

In general, only symptomatic supportive treatment is performed in CD chronic phase, while patients with CCC are recommended to follow the treatment protocol for heart failure according to cardiac commitment grade (72), being heart transplantation the only course of action in case of advanced heart failure (45). Whereas, to patients with digestive involvement, conservative or even surgical treatment is indicated depending on the stage of the disease (73). Regarding the chronic asymptomatic patients, careful follow-up is indicated, with the use of antiparasitic drug therapy recommended in particular cases, such as childbearing-aged women, where treatment can prevent congenital infection and in some cases the development of heart disease (66, 74).

## Classical Endemic Areas for CD

The ancestral lineages of *Trypanosoma cruzi* were probably introduced to South America via bats ~7–10 million years ago (75). The oldest evidence of *T. cruzi* infection came from the detection of parasite DNA in a 9,000-year-old Chinchorro mummy from the coastal area of Atacama Desert (28). It has been hypothesized that Chinchorro people that used to have a nomadic lifestyle participate in the sylvatic cycle of *T. cruzi*. Gradually after their settlement, a domestic cycle of *T. cruzi* transmission emerged (28, 76), which was facilitated by the ability of the vectors to adapt easily to more opened vegetation (77). Additionally, historical findings suggest that many pre-Hispanic cultures were in close contact with triatomine insect vectors in their dwellings before the arrival of European conquerors to South America, Central America, and Mexico (28). From the beginning of the Sixteenth century, there is evidence that CD was present in Latin America, affecting indigenous people as well as the European travelers (78, 79). Some centuries after, in 1908, during an anti-malaria campaign in support of the construction of a railway track in the state of Minas Gerais (Brazil), a railroad engineer warned Carlos Chagas of large blood-sucking insects which lived in local dwellings and bit sleeping people preferentially in the face (12). Then, Chagas dissected them and found numerous trypanosomes in their hindgut (80). At that time, *T. cruzi* transmission cycles were restricted to the sylvatic environment, being initially an enzootic phenomenon, but due to rural exodus, deforestation and urbanization, CD became an anthropozoonosis (81).

After its discovery, CD has remained for many decades as an exclusively rural disease associated with social aspects of poverty in areas of Latin America (40). Indeed, CD was always associated with regions presenting severe political deformation, economic instability, illiteracy and miserable huts (82). The classical endemic area of CD ranges from southern region of the USA to the north part of Argentina and Chile, comprising 21 countries (Argentina, Belize, Bolivia, Brazil, Chile, Colombia, Costa Rica, Ecuador, El Salvador, French Guyana, Guatemala, Guyana, Honduras, Mexico, Nicaragua, Panama, Paraguay, Peru, Suriname, Uruguay, and Venezuela) (40). In this area, about 6 million people are affected, occurring approximately and 28,000 new cases of CD and 12,000 deaths per year (4). The Pan American Health Organization (PAHO) estimates that 65 million people live in areas of exposure and are at risk of being infected (Table 1).

**Table 1:** Estimated number of infected individuals and people at risk of infection in Latin America from 1980 to 2010.

	**1980–1985**		**2005**		**2010**	
	**Infected individuals**	**Individuals at risk of infection (%)**	**Infected individuals**	**Individuals at risk of infection (%)**	**Infected individuals**	**Individuals at risk of infection (%)**
**SOUTHERN CONE**						
Argentina	2,640,000 (10%)	23	1,600,000 (4.1%)	23	1,505,235 (3.64%)	5.42
Bolivia	1,300,000 (24%)	32	620,000 (6.8%)	32	607,186 (6.1%)	5.9
Brazil	6,180,000 (4.2%)	32	1,900,000 (1%)	32	1,156,821 (0.6%)	13.4
Chile	1,460,000 (16.9%)	63	160,200 (1%)	63	119,660 (0.7%)	0
Paraguay	397,000 (21.4%)	31	150,000 (2.5%)	31	184,669 (2.13%)	19.6
Uruguay	37,000 (3.4%)	33	21,700 (0.7%)	33	7,852 (0.23%)	0
**ANDEAN INITIATIVE**						
Colombia	900,000 (30%)	11	436,000 (1%)	11	437,960 (0.95%)	10.5
Ecuador	30,000 (10.7%)	41	230,000 (1.7%)	47	199,872 (1.38%)	28.9
Peru	621,000 (9.8%)	39	192,000 (0.7%)	12	127 282 (0.43%)	4.5
Venezuela	1,200,000 (3%)	72	310,000 (1.2%)	18	193,339 (0.71%)	3.8
**CENTRAL AMERICA**						
Belize	–	–	2,000 (0.7%)	50	1,040 (0.3%)	22.3
Costa Rica	130,000 (11.7%)	45	23,000 (0.5%)	23	7,667 (0.16%)	5.2
El Salvador	900,000 (20%)	45	232,000 (3.4%)	39	90,222 (1.3%)	15.9
Guatemala	1,100,000 (16.6%)	54	250,000 (2%)	17	166,667 (1.2%)	10.3
Honduras	300,000 (15.2%)	47	220,000 (3.1%)	49	73,333 (9.2%)	14.6
NICARAGUA	–	–	58,600 (1.1%)	25	29,300 (0.52%)	11.5
Panama	200,000 (17.7%)	47	21,000 (0.01%)	31	18,337 (5.2%)	13.1
Mexico	–	–	1,100,000 (1%)	28	876,458 (7.8%)	20.9
^*^Guianas/Suriname	–	–	–	–	12,600 (0.8%)	25.1
Total	17,395,000 (4.3%)	25	7,694,500 (1.4%)	20	5,742,167 (1.1%)	12.9

* *Guiana and French Guiana. Source: Adapted from (3, 71)*.

The distribution and epidemiological characteristics of CD can change according to environmental factors and degree of human interference in the wild ecotope. Thus, Coura et al. (42) divided CD in the Americas into four groups based on epidemiological characteristics (the domestic, peridomestic and sylvatic cycle of the parasite) and characteristics of the infection and the disease. The first group includes Venezuela, Peru, Paraguay, Ecuador, Chile, Brazil, Bolivia, and Argentina, where heart disease is predominant, and the wild, peridomestic and domestic cycles are found. In these countries, the parasite transmission by blood transfusion and by *T. infestans* is under control. In the second group, formed by Costa Rica, Colombia and Mexico, the domestic and peridomestic cycles are found with unsatisfactory vector control. In this group, the prevalent form of CD is the chronic chagasic cardiomyopathy. The third group includes El Salvador, Guatemala, Nicaragua, Panama, and Honduras, and is characterized by the presence of the domestic, peridomestic and wild cycles. The information about clinical forms of CD in these countries is very limited. In the fourth group, that includes United States of America (USA), Guyana, French Guyana, Haiti, Jamaica, Suriname, Cuba, Belize, and the Bahamas. In these countries, human cases occur mainly among immigrants from endemic areas, where the wild cycle is predominant (42, 83).

The assumption of vector controlling as the most effective method for preventing *T. cruzi* transmission in endemic areas motivated, in 1991, the establishment of the “Southern Cone Initiative”. This initiative was a multi-country program in the Southern Cone countries (Argentina, Brazil, Bolivia, Brazil, Chile, Paraguay, and Uruguay) which aimed the elimination of *Triatoma infestans*. In the following years, similar programs were also created in endemic areas as the “Initiative of the Andean Countries” (1997), “Initiative of Central America and Mexico” (1998), and “Initiative of the Amazon Countries” (2004) (35). As a consequence of these programs, a marked decrease was observed in the number of cases transmitted by the vector, which also contributed indirectly to a reduction in the infections via blood transfusion and maternal-fetal route (42). The vectorial and blood transfusion transmissions were declared interrupted in Uruguay in 1997, in Chile in 1999, and in Brazil in 2006, decreasing by 70% the incidence of *T. cruzi* in South America (84). However, due to the imbalance caused by environmental and biodiversity changes associated with the presence of human activities close to the sylvatic cycle of *T. cruzi*, the oral transmission has emerged in highly endemic areas such as the Amazon Basin and also in regions where triatomine domestic and peridomestic control has been effective (50). Two foodborne outbreaks occurred in Brazil, one in 2005 in Santa Catarina state (area with vector control), when 24 people were infected after drinking sugarcane juice contaminated with *T. cruzi* (85); and the other in 2006, in Pará state (highly endemic area), with 178 cases of acute disease after eating contaminated açaí fruit (86).

## The New Scenario of Chagas Disease

While the prevalence of CD in Latin America has been reduced in recent decades, a dramatic increase in the number of CD cases in non-endemic countries have been observed, turning the disease into a worldwide public health concern (9, 87).

Human migrations have been indicated as the critical factor for the emergence of CD in areas where it was not previously described (87, 88). In 2017, people born in Latin America and the Caribbean represented the second largest group of international migrants, just behind Asia, with 32 million people living outside their region of birth (89). Of these, the majority was living in Northern America (26 million) and Europe (5 million) (89). The United States are the primary recipients of Latin-American migrants, however, since 2001, when visa regimes to the U.S. became more restricted, Europe is also receiving substantial numbers of immigrants (90, 91).

**Figure 3:**
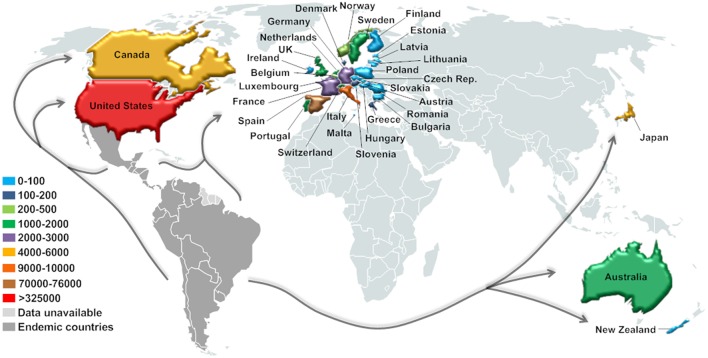
Current estimated number of immigrants with *T. cruzi* infection in non-endemic countries. Estimation based on data for the United States (2007) and Canada (2006) (93), Europe (2008–2011) (94), Japan (2007) (95), Australia (2011), and New Zealand (2006) (92).

In this context, CD has already been detected in non-endemic countries from North America (Canada and the U.S.), Europe (mainly Spain), and the Western Pacific Region (Australia, New Zealand, and Japan) (90, 92) (Figure 3). Currently, around 14% of the $7.2 billion/year estimated global costs with CD (health care and disability-adjusted life-year burden, mainly due to cardiovascular disease) comes from non-endemic countries, and about 12% of these costs emanate from the U.S. and Canada (10). However, the real significance and public health implications of CD in this new epidemiological scenario are still unclear.

Several authors estimate the number of CD cases based on the total number of immigrants received by each host country and the *T. cruzi* prevalence in the country of origin according to the Pan American Health Organization (PAHO) (90, 94, 96–99). On the other hand, results from systematic reviews and meta-analysis seem to indicate more accurate prevalence estimates of CD in non-endemic countries (88, 100). Regardless of the estimate employed, the results indicate a growing number of CD cases in non-endemic countries and therefore the requirement for more attention and efforts toward disease control.

### CD Prevalence in the United States and Canada

The oldest known case of *T. cruzi* infection in the U.S. was confirmed in a mummy dated to 1,150 B.P. (29), but the first CD case in the country was only reported in Texas in 1955 (101). Many southern U.S. states have reported the presence of triatomines, *T. cruzi*, and infected mammalian hosts. However, only 28 human cases of domestically acquired vector-borne CD have been confirmed from 1955 to 2015 (102). This low rate of vector-borne transmission might be the result of lower transmission efficiency of North American vectors as well as better housing conditions (97). Thus, the vast majority of CD cases in the U.S. are from immigrants, who acquired *T. cruzi* in their home countries.

In 2005, it was estimated that more than 22 million people born in CD endemic countries of Latin America were living in the U.S., of which 300,167 were estimated to be infected with *T. cruzi* (97). Mexico contributed the highest number of immigrants with CD (58%), followed by El Salvador (16.4%) and Guatemala (6.8%). Considering that a proportion of 10–15% of infections will develop clinical disease, it is expected that 30,000–45,000 of those infected individuals may have undiagnosed Chagas cardiomyopathy (97). A similar study performed in 2007 estimated a higher number of infected immigrants in the U.S., with about 325,671 potentially infected, of which 20% (65133) have or will have symptoms of CD (93). Inversely, a recent estimate conducted in 2012 indicate a lower prevalence of immigrants with *T. cruzi* infection in the U.S. (238091). Nevertheless, it does not include undocumented immigrants who may represent about 109,000 additional cases (96). In this study, states with the highest estimated numbers of cases are California (30%), Texas (16%), Florida (8%), and New York (7.3%) (96).

The number of international migrants worldwide has continued to overgrow. In 2017, the Department of Economic and Social Affairs of the United Nations reported that around 26 million of Latin America and Caribbean migrants were living in Northern America (89). Thus, it is thought that the number of immigrants with CD living in the U.S. might be even more significant.

A total of 5,553 (3.5%) of the 156,960 Latin American immigrants living in Canada in 2006 were estimated to be infected with *T. cruzi* (93). Of these, the vast majority came from Colombia (1,293), followed by Argentina (968) and El Salvador (913). It is expected that about 1,111 of those immigrants may need medical attention due to CD (93).

### CD Prevalence in Europe, Australia, New Zealand, and Japan

Currently, as well as in the U.S., there is a large number of immigrants living in Europe, with around 5 million people from Latin America (89), most of them in Spain, Italy, France, United Kingdom, and Switzerland (103).

In 2009, it was estimated that about 68,318 to 123,078 immigrants living in Europe were infected by *T. cruzi*, the great majority (ca. 48 million) in Spain (104). Bolivia contributed with the highest number of immigrants with CD (56.4%), followed by Ecuador (11.2%), and Argentina (10.4%). Despite this estimate, only 4,290 confirmed cases were reported until 2009 in Europe, meaning that 94–96% of cases may remain undiagnosed (99). Several other studies estimated the prevalence of CD in Europe, however, results are occasionally quite divergent, mainly due to different sources of the number of Latin American immigrants as well as infection rates (90, 93, 103, 105–107). More recently, a study assembled those estimates arriving between 13,932 and 181,181 cases of CD in Europe with 54,354 immigrants expected to develop CCC (94). Besides that, results from systematic reviews and meta-analysis indicate an underestimation of CD prevalence in some European countries (88, 100). The pooled prevalence of documented cases of CD in the literature in migrants from Bolivia and Paraguay living in Europe (18 and 5.5%, respectively) was higher compared to PAHO prevalence in the countries of origin (6.75 and 2.54%, respectively) which may reflect a higher proportion of migrants from highly endemic areas from Bolivia and Paraguay to Europe. The authors even indicate those estimates to measure the burden of CD in European countries (100).

Japan is home to more than 370,000 Latin American immigrants, most of them from Brazil (87%), with an estimated prevalence of over 4,000 cases of *T. cruzi* infected residents as of 2007 (95). Despite this estimate, only 8 cases of CD have been documented in the period between 1995 and 2015 (95, 108–113). In 2011, a total of 116,430 immigrants from CD endemic countries were residing in Australia, 1,928 (2%) of which had CD. Argentina (*n* = 550), El Salvador (*n* = 366), and Chile (*n* = 280) were the main source countries (62%) of immigrants with CD (92). According to an estimation performed in 2006, New Zealand hosted 82 *T. cruzi* infected residents in a total of 3,615 Latin American immigrants, mostly from Bolivia, Brazil, and Chile (92).

### Blood Transfusion, Congenital, and Post-transplant *T. cruzi* Infection

In non-endemic countries, *T. cruzi* transmission may occur through blood transfusions and organ transplants from infected donors as well as congenital transmission from mother-to-child during pregnancy. Newly acquired *T. cruzi* infections by those routes have been reported in non-endemic areas such as U.S, Spain, Switzerland, and, most recently, Japan (96, 103).

Widespread screening of blood donations for *T. cruzi* infection was implemented in the U.S. in January 2007 and now covers 75–90% of the blood supply (9, 114). Most centers screen all blood donations; however, a small number perform serological tests only for donations from individuals who reported being at risk (such as those from endemic countries, temporary resident, and/or traveling in endemic areas) (90). Since 2007, the American Association of Blood Banks (AABB) has reported 1,908 confirmed cases of *T. cruzi* infection identified through screening of blood donations, the majority of them in the states of California (707), Florida (260), and Texas (176) (96). The proportion of blood donors that are infected with *T. cruzi* is higher in cities with large numbers of Latin American immigrants, such as Los Angeles (1/7,500) and Miami (1/9,000) (115). In Europe, a systematic screening of at-risk blood donations for *T. cruzi* infection was first implemented in the United Kingdom (1999) followed by Spain (2005), France and Sweden (2009), and more recently in Switzerland (2012), and Belgium (2013) (103). The highest rates of positive serology were observed in Italy (3.9% of 128 blood donors) (116), Spain (1.91% of 1,201) (117), France (0.31% of 972) (118), Switzerland (0.08 of 1,183) (119), United Kingdom (0.007% of 38.585) (120), while no case was observed in the Netherlands (0% of 1,333) (121). Since 2003 Australia has tested 154 donors at risk of CD, with an estimated risk of *T. cruzi* transmission of 0.04% (92). Japan has still not implemented routine test-based screening for donated blood to detect *T. cruzi* infection, although a questionnaire is used to determine the self-reported risk to CD (109).

Mother-to-child transmission is another way of *T. cruzi* infection that is of concern in non-endemic countries. *T. cruzi* prevalence in a study performed in 1,350 Latin American pregnant women in Spain was 3.4%, of which 91% came from Bolivia (122). In Texas, the U.S., a study performed in a hospital showed that 10 of 4,000 mothers (0.25%) presented *T. cruzi* infection; most of the women were from Latin America (123, 124). Annually, the estimate of babies with congenital *T. cruzi* infection is between 63 and 315 in the U.S. and 20 to 183 in Europe [80, (104)]. Given that at birth most of the infected newborns are asymptomatic or present non-specific CD symptoms such as low birth weight, respiratory distress, and myocarditis, it is believed that the congenital CD is under-diagnosed (103, 125). Since newborns usually present high rates of parasitemia, congenital infection can be confirmed by direct observation of *T. cruzi* trypomastigotes under microscopy in samples from the cord or peripheral blood (104). In Japan, around 30 newborns were estimated to be infected in the past decade, however, no country in the Western Pacific region present screening programs for *T. cruzi* infection in pregnant mothers and newborns (92, 109).

Organ transplantation has opened another route of *T. cruzi* transmission in non-endemic countries. Five cases of CD after organ transplantation were described in the U.S. (126, 127). Moreover, 17 organs being considered for transplantation in the U.S. were discarded due to seropositive test for *T. cruzi* in 2008 (128). In Spain, *T. cruzi* transmission by cord blood transplants and bone marrow have been documented (129, 130). Also, recently, a case of *T. cruzi* transmission by liver transplantation was reported in a Spanish woman who received the organ from a Bolivian woman donor (131).

## Future Implications

Although in 2019 marks 110 years since the discovery of CD, it is still one of the most important neglected tropical diseases (132). Moreover, with globalization, CD has become a concern in nonendemic countries (133). Taking that into account, the WHO launched in 2007 the Global Network for Chagas Elimination to coordinate global efforts toward CD elimination. Since then, the WHO has conducted a series of meetings: “Control and prevention of Chagas disease in Europe (2009),” “Informal Consultation on Chagas Disease in the Western Pacific (2011),” and the “World Health Assembling resolution Chagas disease: control and elimination (2010)” (134). The non-endemic countries health politics agreed in contributing to control and to interrupt disease transmission by (i) systematically screening blood for transfusions and organs for transplantation, patients under treatment and newborns infected through congenital transmission; (ii) improving clinical diagnosis and case management; (iii) sharing information about CD, and (iv) training health personnel to facilitate diagnosis and medical care (133, 135).

However, the main challenges found to control and treat CD in non-endemic countries are: funding for healthcare education programs; screening programs for pregnant women and donors (blood and organs); access to healthcare for chronically infected individuals; socioeconomic factors; cultural and language barriers faced by immigrants; as well as the lack of information and trust in government programs for immigrants (133). At the same time endemic countries need to overcome the following challenges against CD: control the main vectors and other species of triatomine bugs which are able to adapt and substitute the main vectors; and interruption of *T. cruzi* transmission by vectorial, blood transfusion, organ transplantation, congenital, or vertical routes (134). Moreover, a projection of the implications of climatic change for 2050 on the geographical distribution of both *Rhodnius prolixus* and *Triatoma infestans* in Venezuela and Argentina suggest that climatic niche approach might contribute to the decreasing trend in the number of new cases of *T. cruzi* human infections per year (136). Also, information, education, and communication programs on CD still need to be strengthened at the community level (134). Thus, although public health authorities worldwide and in Latin America have made efforts to control the several forms of transmission of CD, there are still many challenges for the elimination of parasites in humans and domestic and wild reservoirs (134, 137).

As described before, only two drugs are available for CD treatment: benznidazole or nifurtimox, both of which present serious side effects (5, 138). Although Benznidazole, the first treatment approved in the US for CD (139), exists for more than 40 years and is the first-line treatment for CD, only in August 2017 the US Food and Drug Administration (FDA) approved this medication for the treatment of children aged 2–12. Alpern et al. (140) highlighted the excessively priced, and consequently difficult access to drugs for neglected tropical diseases in the US, which might occur with benznidazole once a private company in the US manufactures it. Meanwhile, some clinical trials such as fexinidazole (a new drug candidate), dosing regimens for the treatment of adult patients with CD, optimization of PCR technique to assess parasitological response for patients with chronic CD, population pharmacokinetics study of benznidazole in children with CD and a proof-of-concept study of E1224 (a new drug candidate) to treat adult patients with CD are being evaluated (141). Additionally, a wide range of vaccine candidates (including whole parasites, purified or recombinant proteins, viral vectors and DNA vaccines) and immunization approaches have been tested over the years as a preventive and potential therapeutic strategy against CD [as reviewed by Beaumier et al. (142)]. However, up to now no safe and potent vaccine for human utilization is available (143). So, more financial support is required to research new drugs, vaccine candidates, and immunization approaches.

Another critical point of concern is the association of chronic CD with numerous comorbidities, such as cardiovascular and metabolic diseases, which have been reported in the last decade. Guariento et al. (144) prospectively evaluated 2,497 CD patients and found that 63.8% had other chronic infirmities, with a higher prevalence (20.6%) of systemic arterial hypertension (SAH), followed by diabetes mellitus (0.4%). Likewise, Pereira et al. (145) reported that 86.6% of patients evaluated had at least one comorbidity associated with *T. cruzi* infection, being the major ones SAH (67%) and dyslipidemia (31.9%). In addition, Kamiji and De Olivera (146) observed hypertensive heart disease, coronary or valvular disease in 29.5% of chronic CD patients, while the coexistence of chagasic cardiomyopathy and other heart disease was observed in 26.5% of patients. The development of cerebrovascular disease associated with SAH (147) and dyslipidemia have also been reported in elderly chagasic patients (145, 147). Additionally, necropsies of 92 elderly CD patients revealed SAH (37%), atherosclerosis (62%) and ischemic heart disease (6.5%) (148), indicating an overlap between these comorbidities and CD. Navarro and collaborators (137) reported 75.7% of dyslipidemia in patients with indeterminate form, suggesting an increased risk of progression to the symptomatic form of CD. Since one of the leading causes of mortality in patients with chronic CD is sudden death, these findings indicate that dyslipidemia and/or atherosclerosis may have a direct influence on patient survival (149). Recently, a meta-analysis demonstrated high mortality for both symptomatic and asymptomatic CD patients when compared to controls. Sudden death, cardiovascular diseases, and heart transplantation were the leading causes of death (150). Thus, more efforts are needed to improve the screening for comorbidities in patients with CD in order to provide interventions to tackle reversible disorders. Furthermore, CD patients must have an adequate medical follow-up to improve patient's quality of life and avoid a more substantial financial impact on the health system.

## Conclusions

In 110 years since its discovery and characterization in Latin America, CD reached a global distribution. Nowadays, CD continues to be a topic of great concern in endemic and non-endemic countries, and the cure or complete eradication of this disease are still some aims to be achieved. Given the current scenario, a multidisciplinary approach is essential to address this challenging disease, in order to achieve better control strategies, development of new diagnostic tools and medications, and investigation and treatment of comorbidities associated with chronic CD.

## Author Contributions

KL and TS participated in the design, coordination, and manuscript writing. FA, LB, FD, and MB participated in the writing of the manuscript. KL and FA developed the graphic design of figures. IM-R and TS revised and edited the final manuscript.

### Conflict of Interest Statement

The authors declare that the research was conducted in the absence of any commercial or financial relationships that could be construed as a potential conflict of interest.
